# Regulating Chemokine–Receptor Interactions through the Site-Specific Bioorthogonal Conjugation of Photoresponsive DNA Strands

**DOI:** 10.1021/acs.bioconjchem.3c00390

**Published:** 2023-10-19

**Authors:** Marleen H. M. E. van Stevendaal, Arjan Hazegh Nikroo, Alexander F. Mason, Jitske Jansen, N. Amy Yewdall, Jan C. M. van Hest

**Affiliations:** † Laboratory of Bio-Organic Chemistry, Department of Biomedical Engineering, Institute for Complex Molecular Systems, Eindhoven University of Technology, 5600 MB Eindhoven, The Netherlands; ‡ School of Biotechnology and Biomolecular Sciences, University of New South Wales, Sydney, NSW 2052, Australia; § Department of Pathology, Radboud Institute for Molecular Life Sciences, Radboud University Medical Center, 6500 HB Nijmegen, The Netherlands; ∥ School of Biological Sciences, University of Canterbury, 8041 Christchurch, New Zealand

## Abstract

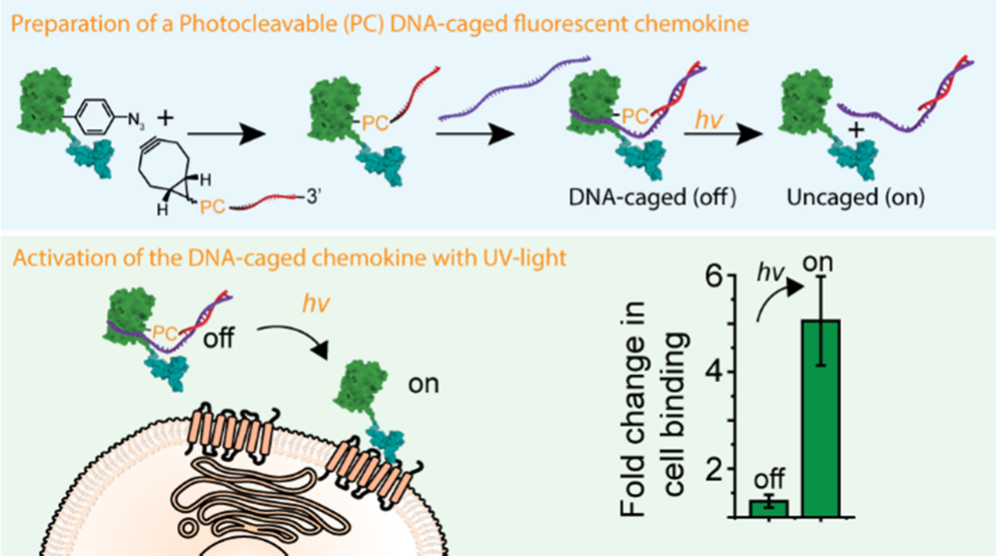

Oligonucleotide conjugation has emerged as a versatile molecular tool for regulating protein activity. A state-of-the-art labeling strategy includes the site-specific conjugation of DNA, by employing bioorthogonal groups genetically incorporated in proteins through unnatural amino acids (UAAs). The incorporation of UAAs in chemokines has to date, however, remained underexplored, probably due to their sometimes poor stability following recombinant expression. In this work, we designed a fluorescent stromal-derived factor-1β (SDF-1β) chemokine fusion protein with a bioorthogonal functionality amenable for click reactions. Using amber stop codon suppression, p-azido-_L_-phenylalanine was site-specifically incorporated in the fluorescent N-terminal fusion partner, superfolder green fluorescent protein (sfGFP). Conjugation to single-stranded DNAs (ssDNA), modified with a photocleavable spacer and a reactive bicyclononyne moiety, was performed to create a DNA-caged species that blocked the receptor binding ability. This inhibition was completely reversible by means of photocleavage of the ssDNA strands. The results described herein provide a versatile new direction for spatiotemporally regulating chemokine–receptor interactions, which is promising for tissue engineering purposes.

## Introduction

In the field of tissue engineering, there is a growing need to better spatially control cell differentiation by employing chemokine gradients. One approach to achieve these gradients is to spatiotemporally regulate when chemokines are available in their active state. They can be reversibly deactivated by blocking their receptor binding site with an inhibitor, which can be removed upon employing a stimulus. Owing to its selectivity, modularity, and programmability, DNA has emerged as a versatile inhibition tool to regulate protein functioning, for example, for biosensing and biomedical applications.^1−3^ Besides noncovalent labeling (e.g., biotin–avidin or coordination chemistry),^4,5^ targeting native functional groups on protein surface residues represents a feasible approach to covalently conjugate DNA to proteins.^6^ This is, however, not trivial for all proteins. The amino acid residues that are usually selected for covalent labeling (e.g., lysines and cysteines) are important for the proper functioning of most chemokines. The N-terminus, and charged residues such as lysine and histidine are, for example, often critical for proper receptor binding and engagement with glycosaminoglycans (GAGs) on cell membranes.^7^ Moreover, disulfide bond formation is crucial to ensure the correct folding. Nevertheless, several methods have been described for the site-specific labeling or modification of chemokines.^8−11^ These strategies include the genetic fusion of a non-native cysteine or peptide tag to the C-terminus for maleimide-cysteine conjugation and enzyme-mediated conjugation, respectively, or the use of Fmoc chemistry near the C-terminus.

State-of-the-art DNA conjugation chemistry is based on bioorthogonal click reactions between modified DNA strands and functional groups on unnatural amino acids (UAAs).^12−15^ Efficient incorporation of UAAs requires the directed evolution of the protein translation system. This is, however, not always straightforward and difficult to extrapolate to proteins that are difficult to express, such as some chemokines. Although UAAs have been successfully incorporated in other growth factors (e.g., insulin-like growth factor 1 and interleukin-4), UAA incorporation in chemokines has to date remained unexplored,^16,17^ even though this could facilitate studies of real-time protein dynamics, and advance the engineering of bioresponsive delivery systems.^18−20^ In addition, performing bioorthogonal chemistry on chemokines, following incorporation of UAAs, circumvents the use of protective groups and enzymes and does not interfere with proper disulfide formation of chemokines.

We have selected the chemokine stromal-derived factor 1β (SDF-1β), also known as CXCL12, as it is widely studied and ubiquitously expressed in many tissues and cell types.^7,21−23^ Moreover, methods for its recombinant expression in *Escherichia coli* (*E. coli*) have been well reported, whereas labeling methods remain limited.^24−28^ SDF-1β plays a crucial role in many physiological processes like inflammation and organogenesis.^7,29^ Belonging to the CXC subfamily of chemokines, SDF-1β contains a characteristic sequence of two N-terminal cysteines separated by one amino acid, indicated by residue X.^30^

Herein, we use an N-terminal fluorescent fusion partner, superfolder green fluorescent protein (sfGFP) as a conjugation site for DNA strands. This provides the chemokine both with fluorescence, often used for probing interactions between chemokines, their cognate receptors, and GAGs, and with a bioorthogonal conjugation handle via an extensively studied UAA incorporation site in GFP.^10,31^ As the conjugation handle is incorporated in the GFP part, no mutations are required for the biofunctional chemokine domain. Furthermore, the positioning of the label is close enough to the functional domain of the chemokine to affect its biological performance.

## Results and Discussion

**Figure 1:**
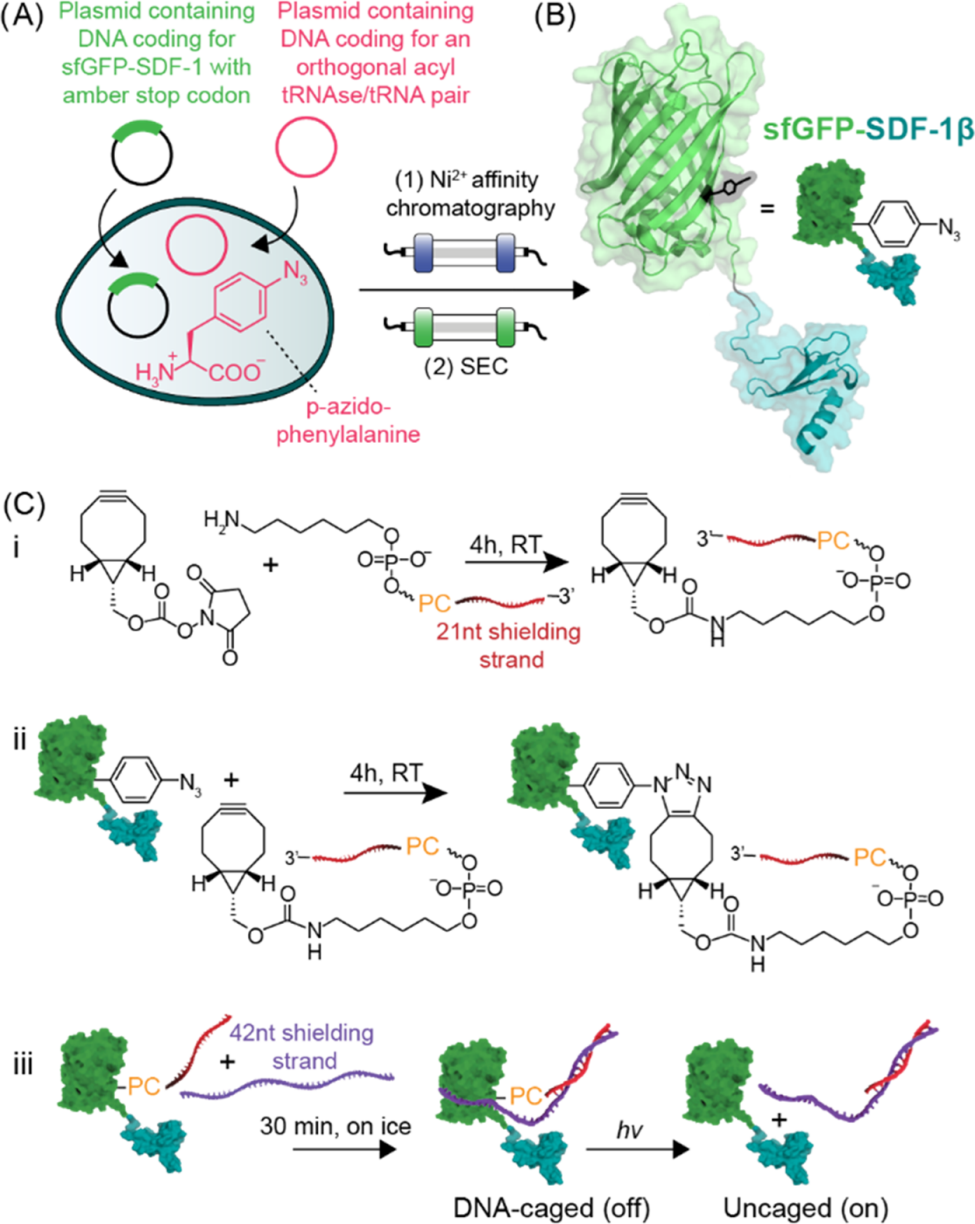
Preparation of the photoresponsive DNA-caged fluorescent chemokine fusion, superfolder green fluorescent protein (green)-stromal-derived factor-1β (cyan) (sfGFP-SDF-1β). (A) Amber stop codon suppression is used to incorporate the unnatural amino acid p-azido-l-phenylalanine (pAzF) at position Y151 in an N-terminal sfGFP domain. The plasmid encoding for sfGFP(Y151pAzF)-SDF-1β is cotransformed in BL21 cells with a plasmid encoding for an orthogonal acyl tRNase/RNA pair. sfGFP(Y151pAzF)-SDF-1β is purified and refolded using Ni^2+^-NTA affinity chromatography and further purified using size exclusion chromatography (SEC). (B) Crystal structure of the fusion protein sfGFP (PDB: 2B3P) and SDF-1β (PDB: 2KEC). (C) Site-specific conjugation of DNA containing a photocleavable 2-nitrobenzyl (PC) linker to sfGFP(Y151pAzF)-SDF-1β. (i) Reaction scheme of the preparation of a bicyclononyne (BCN)-modified single-strand (ss)DNA using *N*-hydroxysuccinimide (NHS) chemistry. (ii) Reaction scheme of the bioconjugation of BCN-modified ssDNA to sfGFP(Y151pAzF)-SDF-1β using strain-promoted alkyne–azide click-chemistry. (iii) Hybridization of a 42nt partial complementary ssDNA strand to 21nt-ssDNA-sfGFP(Y151pAzF)-SDF-1β to create 63nt-DNA-sfGFP(Y151pAzF)-SDF-1β, followed by photocleavage using UV light.

First, an amber stop codon was incorporated at position Y151 of sfGFP, and *E. coli* cells were cotransformed with a plasmid encoding for sfGFP(Y151X)-SDF-1β and a plasmid encoding for an orthogonal acyl tRNase/RNA pair (pEvol) for the incorporation of the UAA p-azido-_L_-phenylalanine (pAzF) (Figure 1A,B). Conjugation of bicyclononyne-modified single-stranded DNAs (ssDNA) to sfGFP(Y151pAzF)-SDF-1β created a DNA-caged chemokine that blocked receptor interactions. Removal of the DNA strands, through the incorporation of a photocleavable linker, made this blockade reversible (Figure 1C).

**Figure 2:**
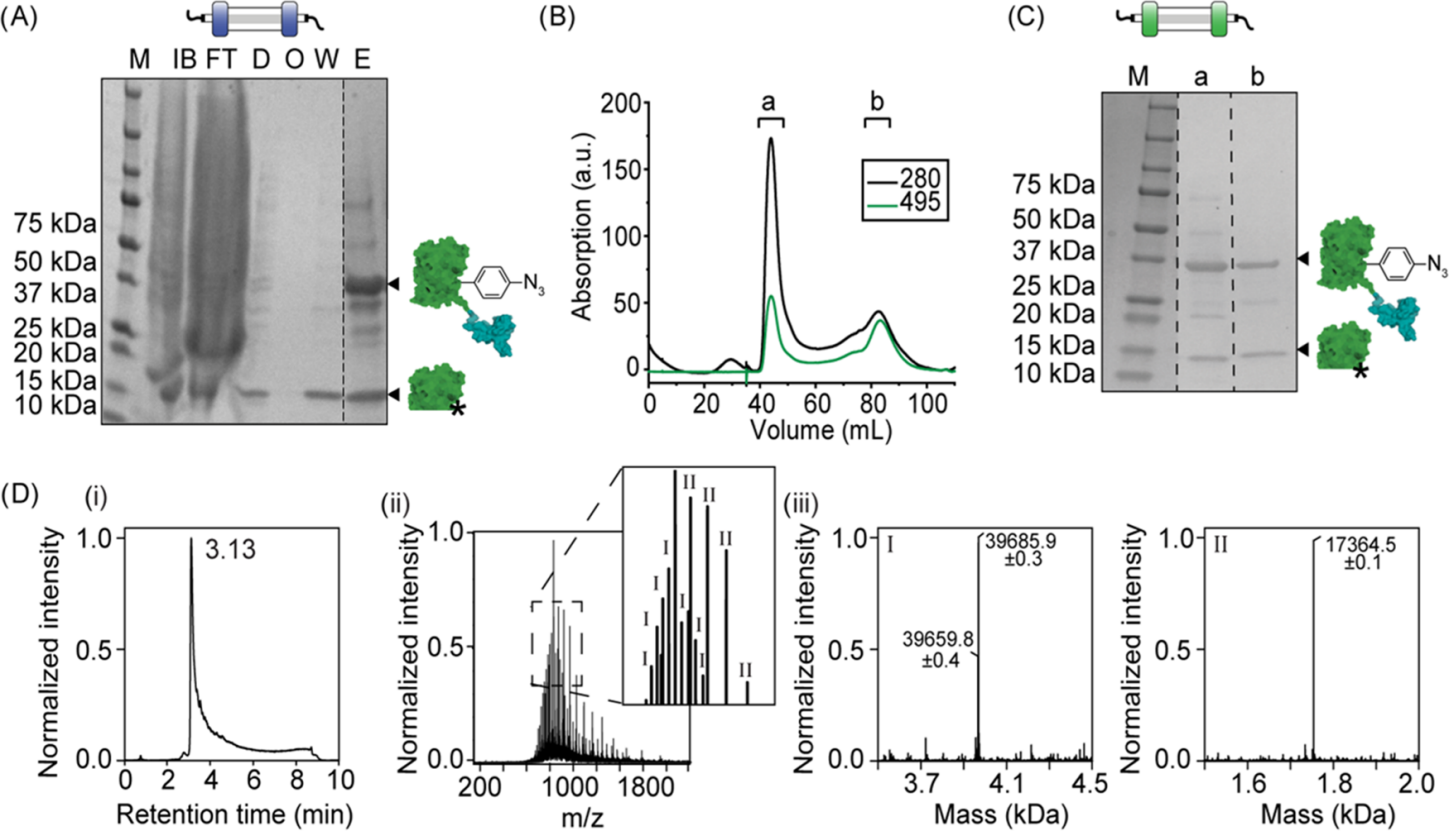
Purification and characterization of sfGFP(Y151pAzF)-SDF-1β following expression from BL21(DE3) cells. (A) SDS-PAGE analysis of the purification and on-column refolding of sfGFP(Y151pAzF)-SDF-1β from inclusion bodies. The protein, containing a C-terminal hexahistidine tag, was unfolded in 6 M guanidinium hydrochloride and loaded onto a column containing Ni^2+^-NTA-agarose resin. On the column, the majority of guanidinium hydrochloride was washed away in the flow through (FT) using a detergent (D) buffer containing Triton X-100 and 2-mercaptoethanol, allowing the protein to fold into its tertiary structure. Next, the disulfide bonds were reformed by washing away the reducing agents using an oxidation (O) buffer. The protein was then washed (W) with 30 mM imidazole and subsequently eluted (E) using 1 M imidazole. Different products are indicated with cartoons: sfGFP(Y151pAzF)-SDF-1β (39.7 kDa) and truncation product sfGFP* (17.4 kDa). (B) Size exclusion chromatogram (SEC) of the pooled elution fractions. Signal was detected at 280 nm (aromatic amino acids) and 495 nm (GFP). (C) SDS-PAGE analysis of the major SEC peaks detected at 495 nm. (D) LC-MS-Qtof analysis. Total ion count chromatogram (i). *m*/*z* spectrum of the signal at 3.13 min detected in the chromatogram (ii). Deconvoluted mass spectra of ion series I and II found in spectrum D (iii). Calculated molecular weight of sfGFP(Y151pAzF)-SDF-1β is 39 686.6 Da, found molecular weight is 39 685.9 ± 0.3 Da. Calculated molecular weight of sfGFP(151Y)-SDF-1β is 39660.6 Da, found molecular weight is 39 659.8 ± 0.4 Da. Calculated molecular weight of truncation product sfGFP* is 17 366.7 Da, found molecular weight is 17 364.5 ± 0.1 Da.

Following the expression of sfGFP(Y151pAzF)-SDF-1β, the majority of protein precipitated in inclusion bodies (Table S2 and Figure S1). For this reason, on-column refolding was used to obtain the protein in its native structure.^25^ After expression and cell lysis by ultrasonic disruption, the protein was unfolded and subsequently refolded on a Ni^2+^-NTA resin (Figure 2A). Besides sfGFP(Y151pAzF)-SDF-1β, a side product of ∼17 kDa (sfGFP*) was copurified. This product could be attributed to a truncated product up to residue 151; this is the site of the amber stop codon, and premature termination at this site, leading to a protein with the observed molecular weight is not unexpected. Both Ni^2+^-NTA affinity chromatography and size exclusion chromatography (SEC) were unsuccessful in removing sfGFP*, suggesting that it interacts with sfGFP(Y151pAzF)-SDF-1β. A possible explanation is the concentration-dependent dimerization of GFP.^32^

It is known that both this chemokine and GFP have the propensity to form multimeric assemblies.^33^ Indeed, sfGFP(Y151pAzF)-SDF-1β eluted as multiple peaks during SEC, the first peak likely being soluble aggregates (Figure 2B,C). Further analysis using nonreducing SDS-PAGE revealed that aggregation in peak a was primarily caused by wrongly formed disulfide bonds (Figure S2A). Native PAGE analysis further confirmed the aggregation in peak a (Figure S2B). Peak b appeared as a mix of monomers and dimers or other higher-order multimers. Only a small fraction of protein that eluted in peak b was aggregated in the absence of a reducing agent. As the presence of aggregates would complicate subsequent modification and biological evaluation, only peak b, eluting around 80 mL, was collected. LC-MS-Q-TOF analysis confirmed the incorporation of pAzF and the formation of two disulfide bonds in sfGFP(Y151pAzF)-SDF-1β (Figure 2D). Approximately one-third of the protein built in a tyrosine in place of pAzF. The data presented here suggest that SDF-1β was successfully expressed when N-terminally fused to sfGFP(Y151PazF), which equips the chemokine with fluorescence for visualization purposes and a site-selective bioorthogonal handle.

**Figure 3:**
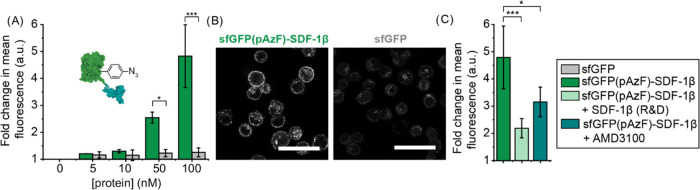
Binding of sfGFP(Y151pAzF)-SDF-1β to its cognate receptors on the cell membrane of HeLa cells. (A) Flow cytometry analysis of the fluorescence of HeLa cells following incubation with different concentrations of sfGFP(Y151(pAzF))-SDF-1β (green) and sfGFP (gray). The binding is represented as a fold change in fluorescence compared to the untreated control. *N* ≥ 2. 10 000 cells were measured per sample. (B) Representative confocal images of HeLa cells incubated with 100 nM sfGFP(Y151pAzF)-SDF-1β and 100 nM sfGFP. Scale bars represent 50 μm. (C) Flow cytometry analysis of the fluorescence of HeLa cells following a competition experiment between 100 nM sfGFP(Y151pAzF)-SDF-1β and 1 μM commercial SDF-1β (R&D) or CXCR4 blocking agent AMD3100. *N* ≥ 3. 10 000 cells were measured per sample. Significance was assessed using Welch’s *t* test. Significance levels are indicated by *p < 0.05, ***p < 0.01.

Next, the receptor binding capacity of SDF-1β was studied when it was fused to sfGFP(Y151pAzF). HeLa cells were chosen for this study as they are known to express high levels of CXCR4 and CXCR7, the cognate receptors for SDF-1β.^34,35^ Flow cytometry analysis revealed that the presence of SDF-1β at the C-terminus of sfGFP increased membrane binding to HeLa cells when compared to sfGFP only (Figures 3A and S3A,B). Confocal analysis revealed that sfGFP(Y151pAzF)-SDF-1β predominantly localized on the cell membrane, but a small fraction was also found intracellularly (Figure 3B).^36^ To study the effect of the fusion partner sfGFP(Y151AzF) on the binding affinity of SDF-1β, the binding profiles of sfGFP(Y151pAzF)-SDF-1β and SDF-1β were compared. sfGFP(Y151pAzF)-SDF-1β bound to the membrane of HeLa cells with a higher affinity than SDF-1β (Figure S4). A possible explanation is that the tendency of sfGFP(Y151pAzF) to form dimers promotes dimerization of SDF-1β, thereby altering its receptor binding properties.^32,33^ This property should be carefully considered when studying the biological functions of these and other sfGFP-fusion proteins.

**Figure 4:**
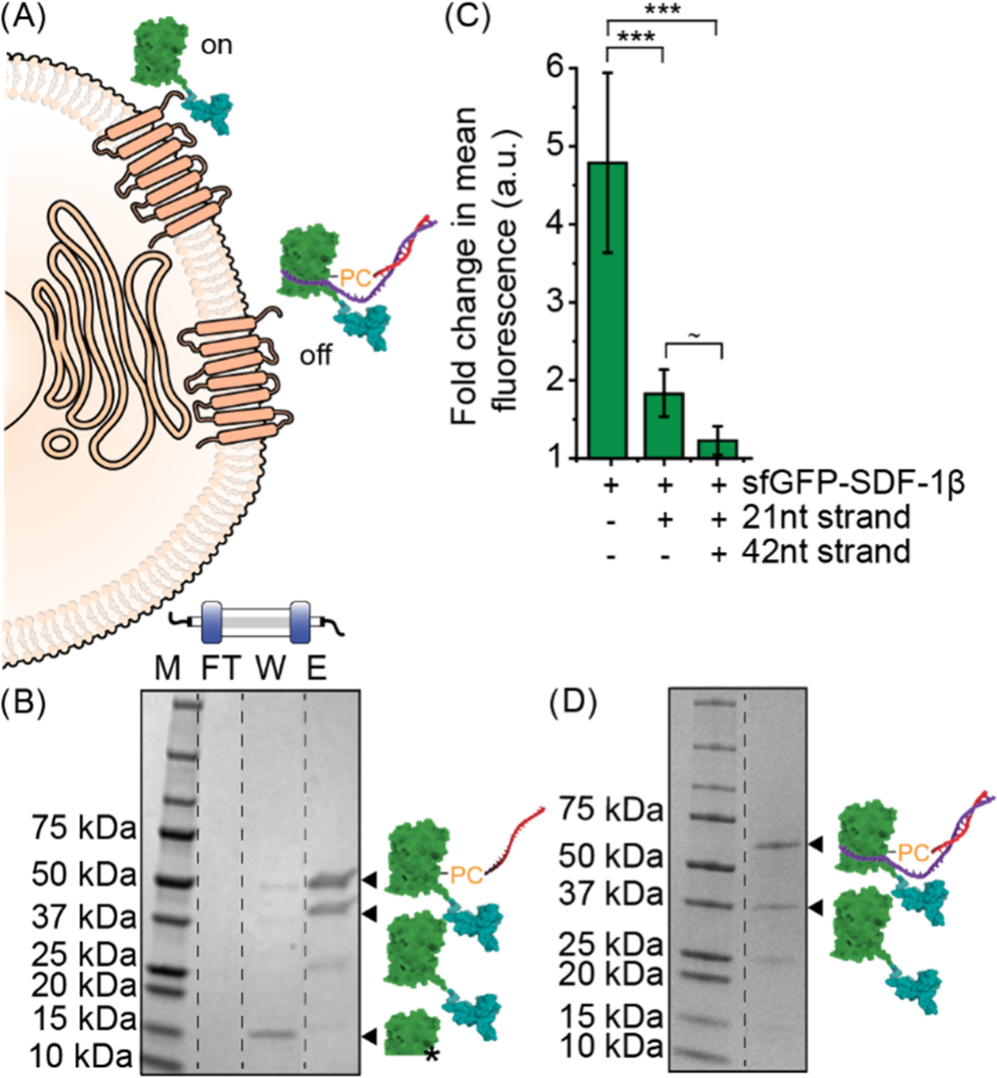
(A) Schematic illustrating the blocked receptor binding following conjugation of DNA strands. (B) SDS-PAGE analysis of the Ni^2+^-NTA affinity chromatography purification of the conjugation reaction between sfGFP(Y151pAzF)-SDF-1β and 21nt-ssDNA containing a photocleavable linker (PC). FT is flow through; W is the wash fraction; and E is the elution fraction. (C) Flow cytometry analysis of the receptor binding of 100 nM sfGFP(Y151pAzF)-SDF-1β on the cell membrane of HeLa cells before and after conjugation to 21nt-ssDNA and hybridization to 42nt-ssDNA strand, when kept in the dark. *N* ≥ 3. 10 000 cells were measured per sample. Significance was assessed using a Welch’s *t* test. Significance levels are indicated by ***p < 0.01 and ^∼^*p* < 0.1 (not significant). (D) SDS-PAGE analysis of 21nt-ssDNA-sfGFP(Y151pAzF)-SDf-1β following hybridization to 42nt-ssDNA.

To study the specificity of the binding, 1 μM commercially expressed SDF-1β and CXCR4 blocking agent AMD3100 were coincubated with 100 nM sfGFP(Y151pAzF)-SDF-1β.^26,37^ Its binding decreased with approximately 50 and 30% following competition from SDF-1β and AMD3100, respectively (Figures 3C and S3C,D). These data indicate that sfGFP(Y151pAzF)-SDF-1β is properly folded and can still interact with its cognate receptors. To demonstrate that the incorporated azide is available and can be used to regulate receptor binding, sfGFP(Y151pAzF)-SDF-1β was reacted with bicyclononyne (BCN)-modified DNA strands (Figure 4A). We first selected a short DNA oligo consisting of 21 nucleotides (nt) based on a design previously reported by our group and studied whether this length would be sufficient to block receptor binding.^38^ 3 equivalents of BCN-modified 21nt-ssDNA were added to 1 equivalent of sfGFP(Y151pAzF)-SDF-1β, after which Ni^2+^-NTA affinity purification was used to remove excess ssDNA (Figure 4B). SDS-PAGE analysis showed that this purification step also removed sfGFP*, indicating that the conjugation of ssDNA disrupted its interaction with sfGFP(Y151pAzF)-SDF-1β, likely due to electrostatics. Approximately 60% of sfGFP(Y151pAzF)-SDF-1β was reacted, which did not increase upon the addition of 5 (59%), or 10 (59%) equivalents of ssDNA. This suggests that conjugation is limited by the incorporation efficiency of p-azido-l-phenylalanine.

Conjugation to 21nt-ssDNA significantly decreased the binding capacity of SDF-1β to the cell membrane of HeLa cells (Figures 4C and S5A). However, we still observed some residual binding. In order to minimize this and force the design toward an on/off system, we tripled the amount of negative charge by the addition of a complementary 42nt-ssDNA strand, adding in total 63nt (Figures 4C,D and S5B,D). The addition of the 42nt-ssDNA strand indeed almost completely blocked receptor binding. Since the chemokine is likely partly dimerized when engaging with its receptors, the difference between conjugation efficiency and binding affinity can be explained by the fact that the receptor interaction is already strongly diminished if one of the two chemokines is modified.^33,39^ Charged residues on SDF-1β are important for engaging with the cell membrane. For example, reduced GAG binding, which is mediated by positively charged residues, is known to diminish receptor binding.^7^ The presence of ssDNA on the surface of sfGFP(Y151pAzF)-SDF-1β likely diminishes cell membrane interactions, thereby minimizing the chances of engaging with CXCR4 and CXCR7. Together, these data show that ssDNA can be site-specifically conjugated to sfGFP(Y151pAzF)-SDF-1β, which results in a loss of receptor binding capacity.

**Figure 5:**
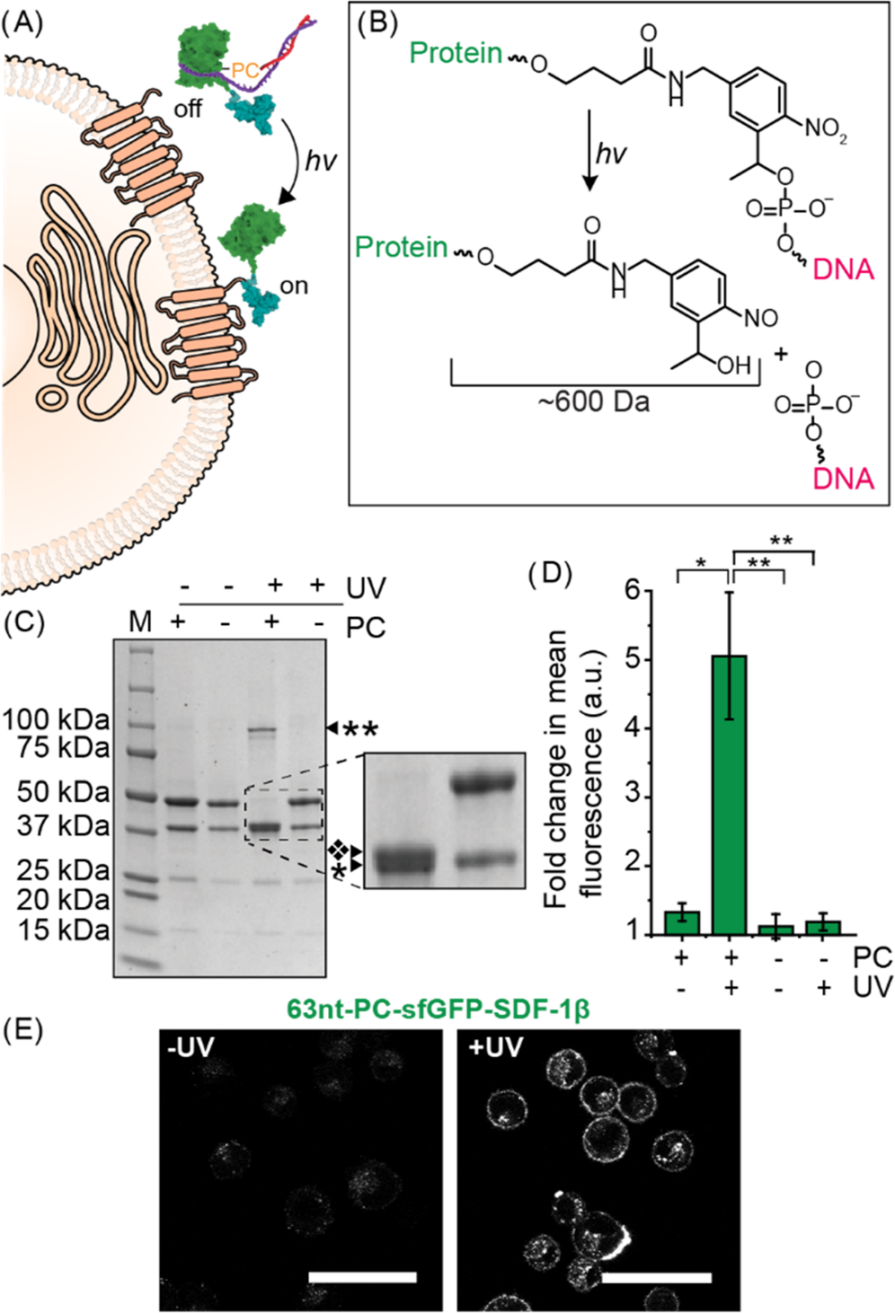
(A) Schematic illustrating the restored receptor binding of sfGFP(Y151pAzF)-SDF-1β following UV photocleavage of the linker (PC) and subsequent removal of DNA strands. (B) Structure of 2-nitrobenzyl photocleavable linker between sfGFP(Y151pAzF)-SDF-1β and the ssDNA strand before and after irradiation with UV light. Part of the linker (∼600 Da) remains attached to the protein. (C) SDS-PAGE analysis of the cleavage of 21nt-ssDNA from sfGFP(Y151pAF)-SDF-1β in the presence or absence of a PC and UV light; * indicates sfGFP(Y151pAF)-SDF-1β monomers, black diamond minus white X indicates a band just above the monomer band; and ** indicates multimers of sfGFP(Y151pAzF)-SDF-1β. (D) Flow cytometry analysis of the receptor binding of 100 nM 63nt-DNA-sfGFP(Y151pAzF)-SDF-1β on the cell membrane of HeLa cells in the presence or absence of a PC and UV light. *N* ≥ 2. 10 000 cells were measured per sample. Significance was assessed using a *t* test. Significance level is indicated by *p < 0.05 and **p < 0.02. (E) Representative confocal images of HeLa cells incubated with 100 nM 63nt-DNA-sfGFP(Y151pAzF)-SDF-1β in the absence or presence of UV light. Scale bars represent 50 μm.

The 2-nitrobenzyl photocleavable linker incorporated between sfGFP(Y151pAzF)-SDF-1β and the 5′ end of the 21nt-ssDNA strand can be cleaved with UV light (300–350 nm) to liberate the DNA-caged chemokine, which is hypothesized to restore receptor binding (Figure 5A,B). The use of photocleavable nitrobenzyl linkers has been demonstrated as a feasible approach to activate or release bioactive molecules such as chemokines.^40−42^ In addition, a photocaged SDF-1α variant has been used to induce the migration of T cells upon UV exposure, indicating that this is a viable method to regulate chemokine activity.^43^ SDS-PAGE analysis indeed confirmed that in the presence of this photocleavable linker, the DNA could be cleaved off within 10 min of UV irradiation, with some side product formation (Figures 5C and S6A). This side product formation is expected to occur during a radical coupling process, as addition of the antioxidant ascorbic acid prevented the formation of these species (Figure S6B) Cleaved sfGFP(Y151pAzF)-SDF-1β ran slightly above the sfGFP(Y151pAzF)-SDF-1β monomers, which is likely caused by the remaining part of the linker still present after photocleavage (+∼600 Da). Cleavage of the 63nt-DNA from sfGFP(Y151pAzF)-SDF-1β fully returned its ability to bind its cognate receptors without the necessity of adding an antioxidant (Figures 5D,E and S5C,E). These data demonstrate that receptor binding of SDF-1β can be regulated by UV-mediated removal of blocking DNA strands.

## Conclusions

In summary, we described a protocol for the successful expression and refolding of the chemokine SDF-1β with a bioorthogonal functionality that can be used to regulate receptor interactions. We fused SDF-1β N-terminally to sfGFP, a robust fluorescent protein in which UAA incorporation has been well described. The refolded fusion protein sfGFP(Y151pAzF)-SDF-1β successfully engaged with its cognate receptors. A DNA-caged variant, where in total 63nt-DNA strands were site-specifically conjugated to the incorporated azide, exhibited impaired receptor binding. Furthermore, integration of a photocleavable 2-nitrobenzyl linker between the ssDNA handle and the fusion protein created a light-responsive system that restored receptor binding once exposed to UV light. Together, the results described in this paper provide a first blueprint for optically controlling chemokine–receptor interactions by employing UAAs as bioorthogonal handles to conjugate DNA activity switches. This concept could be adopted in future studies to regulate spatiotemporal interactions between SDF-1β and a scaffold, with the ssDNA acting as a selective handle that provides spatial control and the photocleavable linker providing temporal control.

## Supporting Information Available

Experimental procedures, DNA sequences, flow cytometry histograms, supporting SDS-PAGE gels, and fluorescence spectra of 63nt-DNA-sfGFP(Y151pAzF)-SDF-1β (PDF)
