# Supplementary material for: Regulating Chemokine–Receptor Interactions through the Site-Specific Bioorthogonal Conjugation of Photoresponsive DNA Strands
Source: Bioconjug Chem. 2023 Oct 19;34(11):2089–95. doi: 10.1021/acs.bioconjchem.3c00390 (PMC10655040; doi:10.1021/acs.bioconjchem.3c00390)
Supplement: Supplementary file 1 — bc3c00390_si_001.pdf [file bc3c00390_si_001.pdf]

Supporting Information

Regulating chemokine-receptor interactions through the site-specific bioorthogonal conjugation of photoresponsive DNA strands

Marleen H.M.E. van Stevendaal,† Arjan Hazegh Nikroo,† Alexander F. Mason,‡ Jitske Jansen, § N. Amy Yewdall,# and Jan C.M. van Hest†*

†Laboratory of Bio-Organic Chemistry, Department of Biomedical Engineering, Institute for Complex Molecular Systems, Eindhoven University of Technology, 5600 MB Eindhoven, the Netherlands

‡School of Biotechnology and Biomolecular Sciences, University of New South Wales, NSW 2052 Sydney, Australia

§Department of Pathology, Radboud Institute for Molecular Life Sciences, Radboud University Medical Center, 6500 HB Nijmegen, the Netherlands

#School of Biological Sciences, University of Canterbury, NZ 8041 Christchurch, New Zealand

Materials and methods

Materials

Solvents and reagents were obtained from commercial sources and used without further purification unless otherwise stated. All amine-modified and dye-labeled ssDNA strands were obtained HPLC-pure from IDT Integrated DNA Technologies, whereas all unlabeled DNA strands were obtained purified by using a desalting column. Upon arrival, all DNA strands were dissolved in MilliQ in DNA Lo binding tubes (Eppendorf) to reach a final concentration of 0.01-1 mM. For cell culture: Dulbecco's Modified Eagle's Medium (DMEM) (#,41965062) fetal bovine serum (FBS) (#26140079), penicillin/streptomycin (#15140122), trypsin-EDTA (0.05%) (#25300054) and Live Cell Imaging Solution (#A14291DJ) were purchased from ThermoFisher Scientific. SDF-1β was purchased from R&D systems (#351-FS-010/CF). AMD3100 (#239825) was supplied by Merck. AlexaFluor647-conjugated-anti-human/mouse SDF-1 antibody was purchased from R&D systems (#FAB350R).

Table S1. ssDNA-handle sequences used in this chapter

Handle ssDNA strand	DNA sequence
Handle PC-21	5'-AmMC6- iSpPC-GGTATCCTTCAACGTCTAGTC-3'
Handle 21	5'-AmMC6-GGTATCCTTCAACGTCTAGTC-3'
Complementary-42	5' -GACTAGACGTTGAAGGATACCCAACCCACAACACACCACCAC-3'

Table S2| Sequences, parameters and yield of the proteins used in this communication. Green: C-terminal hexahistidine tag. Blue: N-terminal lysine and proline of SDF-1β. X (Red): non-canonical amino acid p-azido-L-phenylalanine. Parameters were calculated using the online ProtParam tool (ExPASy).

**Protein**	**Sequence**	**Mass (kDa)**	**Yield (mg L[1])**
sfGFP	MVKMGASKGEELFTGVVPILVELDGDVNGHKFSVRGEGEGDATNGKL TLKFICTTGKLPVPWPTLVTTLTYGVQCFSRYPDHMKQHDFFKSAMP EGYVQERTISFKDDGTYKTRAEVKFEGDTLVNRIELKGIDFKEDGNILG HKLEYNFNSHNVYITADKQKNGIKANFKIRHNVEDGSVQLADHYQQ NTPIGDGPVLLPDNHYLSTQSALSKDPNEKRDHMVLLEFVTAAGITHG MDELYKTLPETGENLYFQSGGSHHHHHH*	29.7	Preparation is described in Ref [1].[1]
sfGFP (Y151pAzF)-SDF-1β	MVKMGASKGEELFTGVVPILVELDGDVNGHKFSVRGEGEGDATNGKL TLKFICTTGKLPVPWPTLVTTLTYGVQCFSRYPDHMKQHDFFKSAMP EGYVQERTISFKDDGTYKTRAEVKFEGDTLVNRIELKGIDFKEDGNILG HKLEYNFNSHNVXITADKQKNGIKANFKIRHNVEDGSVQLADHYQQ NTPIGDGPVLLPDNHYLSTQSALSKDPNEKRDHMVLLEFVTAAGITHG MDELYKTLPETGGGSHMGGSDDDDKKPVSLSYRCPCRFFESHVARAN VKHLKILNTPNCALQIVARLKNNNRQVCIDPKLKWIQEYLEKALNKRF KMGGSENLYFQSGGSHHHHHH*	39.7	**Purified from** **Soluble:** Total: 0.06 **Inclusion bodies:** Total: 0.59 Peak b: 0.52

Methods

DNA molecular biology and cloning

All gBlocks were obtained from IDT. The sequence of sfGFP was copied from Altenburg et al.1, and the sequence for SDF-1β was obtained from R&D systems (351-FS-010/CF, UniProt accession number: P48061). The linker was designed to be flexible using residues that not readily form tertiary structures to ensure that the N-terminal lysine and proline of SDF-1β are not blocked when engaging with its receptors. In addition, should it be required, sfGFP could be cleaved from SDF-1β by the incorporation of a cleavage site for enterokinase. All sequences were optimized using the IDT Codon Optimization Tool for Escherichia coli (E. coli) based on the amino acid sequence. The vector pET28a and gblocks were both digested using the restriction enzymes NcoI and XhoI (New England Biolabs). Following ligation, the constructs were verified using Sanger sequencing (BaseClear).

sfGFP(Y151pAzF)-SDF-1β expression and purification

pET28a encoding for sfGFP(Y151pAzF)-SDF-1β was co-transformed into *E. coli* BL21(DE3) bacterial cells with a pEVOL plasmid containing an engineered amino acyl tRNAse/tRNA pair from *M. jannaschii* (a kind gift from Peter Schultz, Addgene plasmid #31186).[2,3] The bacteria were cultured at 37 °C in 1 L terrific broth (TB) medium, 25 µg mL^{-1} kanamycin and 25 µg mL^{-1} chloramphenicol. Protein expression was induced at OD600=0.6 by addition of 1 mM IPTG and 0.02% (w/v) arabinose. Simultaneously, p-azido-L-phenylalanine was added directly to the culture medium at a final concentration of 1 mM (Iris Biotech). Expression was carried out in the dark for ca 16 hrs at 30 °C. Purification of sfGFP(Y151pAzF)-SDF-1β was based on previously reported methods.[4] The cells were harvested by centrifugation at 4000 xg for 10 min at 4 °C and either resuspended in resuspension buffer (50 mM sodium phosphate (Na_2HPO_4) pH 7.4, 300 mM NaCl, 10 mM imidazole, 1 mM phenylmethylsulfonyl fluoride, and 0.1% (v/v) 2- mercaptoethanol) when lysed immediately, or in LB when flash-frozen in liquid N_2 and subsequently stored at -20 °C. The cells were lysed by ultrasonic disruption 7 times for 30 seconds at 70% amplitude (Branson Sonifier 150), after which the lysate was centrifuged at 15,000 xg for 45 min at 4 °C. The supernatant, which contained the soluble expressed protein, was collected, and is further referred to as the soluble fraction. The pellet, which contained the protein that aggregated as inclusion bodies, was dissolved in solubilization buffer (50 mM sodium phosphate (Na_2HPO_4) pH 7.4, 300 mM NaCl, 10 mM imidazole, and 6 M guanidinium hydrochloride) and incubated at 4 °C. After 30 min, the suspension was centrifuged at 15,000 xg for 45 min and the supernatant, herein further referred to as the inclusion body fraction, was collected. sfGFP(Y151pAzF)-SDF-1β from both the soluble fraction and inclusion body fraction was further purified using Ni^{2+}-NTA affinity chromatography employing the C-terminal hexahistidine tag. The fractions were loaded onto 1 mL Ni^{2+}-NTA agarose resin (Qiagen) and in the case of the soluble fraction, the resin was washed with 4 times two column volumes wash buffer (20 mM Tris pH 8.0, 30 mM imidazole, 500 mM NaCl) after which the protein was eluted using elution buffer (25 mM HEPES pH 7.4, 300 mM NaCl, 1 M imidazole). In the case of the inclusion body fraction, the column was washed with 20 mL of detergent buffer (20 mM Tris pH 8.0, 100 mM NaCl, 1% Triton X-100 (v/v), 10 mM 2-mercaptoethanol), and then 20 mL oxidation buffer (20 mM Tris pH 8.0, 100 mM NaCl, 5 mM β-cyclodextrin, 1 mM reduced glutathione, 0.5 mM oxidized glutathione) prior to the washing and elution step. Elution fractions containing sfGFP(Y151pAzF)-SDF-1β were pooled and buffer exchanged to 25 mM HEPES pH 7.4, 300 mM NaCl buffer (Amicon Ultra-4 10 kDa MWCO, Millipore, or Slide-A-lyzer MINI 20 kDa MWCO, Thermo Scientific) prior to further purification using size exclusion chromatography (HiLoad® 16/600 Superdex® 200 pg, GE Healthcare). The eluted fractions were analyzed using SDS-PAGE and

Coomassie Brilliant Blue staining, and the fractions eluting in different peaks were pooled and concentrated. Protein concentrations were determined using the 485 nm absorbance determined on a ND-1000 spectrophotometer (ThermoScientific) and the theoretical extinction coefficient of sfGFP: 83,300 M^{-1} cm^{-1}.[5] The protein was flash-frozen in liquid N_2, and stored at –80 °C for further use.

Native PAGE

Samples were prepared by mixing the protein (ca 2 μg) with 4x native PAGE loading buffer (248 mM Tris·HCl, glycerol 40 % (v/v), bromophenol blue 0.02 % (w/v)), after which the samples were loaded on the gel (4–20% Mini-PROTEAN TGX Precast Protein Gel, Bio-Rad). The gel was run at 4 °C with pre-cooled native PAGE running buffer (25 mM Tris·HCl, 192 mM glycine) containing a cooling pack for 3 hrs at 100 V in a cold room. Subsequently, the gel was shortly washed for 5 minutes in demineralized water and fluorescence was imaged using an ImageQuant 350 gel imager. Afterwards, the gel was stained with coomassie blue staining solution (BioRad), destained in demineralized water and again imaged.

Mass spectrometry

The mass of sfGFP(Y151pAzF)-SDF-1β was confirmed using a High-Resolution Liquid Chromatography system (ACQUITY UPLC I-Class, Waters) coupled to a Quadrupole Time of Flight mass spectrometry system (Xevo G2, Waters). Protein samples were buffer exchanged to MilliQ supplemented with 0.1% v/v formic acid before injection. Proteins were separated on a reverse phase column (Polaris 3 C8-A 100 x 2.0 mm or C18-A 150x2.0 mm, Agilent) using an acetonitrile gradient in MilliQ supplemented with +0.1% v/v formic acid. Deconvoluted mass spectra were acquired using the MaxEnt1 algorithm in Masslynx 4.1 (Waters).

Preparation of BCN-labeled ssDNA

Functionalization of 5'-amine modified ssDNA with (1R,8S,9s)-bicyclo[6.1.0]non-4-yn-9-ylmethyl N-succinimidyl carbonate (BCN-NHS) was performed by adding 3 equivalents of BCN-NHS (10 mM stock solution in dry DMSO) to 1 equivalent 5'-amine modified ssDNA (1 mM of in 1x PBS, pH 7.2) and incubating for 4 h at room temperature under continuous shaking at 800 rpm. Unreacted BCN-NHS was removed by ethanol precipitation by the direct addition of 5 M sodium chloride solution and ice-cold 100% ethanol to the reaction mixture. The mixtures were incubated for at least 30 min at –30 °C. After centrifugation (14,000 xg for 30 min at 4 °C) the pellet, containing the ssDNA, was collected and dissolved in 100 μL of 1x PBS, pH7.2. This procedure was repeated once. The pellet was washed once more with 95% ice-cold ethanol (v/v %, in MilliQ), after which the mixture was centrifuged (14,000 g for 15 min at 4 °C) and the pellet was lyophilized and stored at –30°C.

Preparation of ssDNA-sfGFP-SDF-1β conjugates

Conjugation reactions between sfGFP(Y151pAzF)-SDF-1β and BCN-labeled-ssDNA (with or without PC) were performed on a 250 μL scale in a 1:3, 1:5 or 1:10 ratio. The reactions were left to react in the dark at room temperature for 4 hrs whilst stirring at 800 rpm in a Lo-DNA binding tube. ssDNA conjugated protein was subsequently purified using Ni^{2+}-NTA chromatography using previously described methods and buffers. The fractions were analyzed using SDS-PAGE and Coomassie Brilliant Blue staining, after which the elution fractions containing the purified product were pooled and the concentration was determined by measuring the absorbance at 485 nm.

UV light irradiation

SsDNA-sfGFP-SDF-1β was exposed to UV light using a UV source (UVL-36, Promed GmbH, 4 x 9 W) for different amounts of time up to 10 min. Protein-ssDNA conjugates (in solution or in coacervates) were always kept on ice. Cleavage was monitored using SDS-PAGE. Fluorescence before and after UV irradiation was monitored using fluorescence measurements on the platereader (Figure S7).

Platereader measurements

Fluorescence scans of (sfGFP(-21nt-ssDNA-Cy3) and 63nt-DNA-PC-sfGFP-SDF-1β were made using a Spark 10 M plate reader (Tecan) or a Safire II (Tecan). Emission was detected between 510-750 nm following excitation at 485 nm.

Receptor binding assays

Cell culture and flow cytometry

HeLa cells were grown as a monolayer in DMEM, supplemented with 10% (v/v) FBS and 1% (v/v) penicillin/streptomycin in a humidified atmosphere of 5% CO_2 at 37 °C. The receptor binding of the recombinant protein sfGFP(Y151pAzF)-SDF-1β was examined using flow cytometry. HeLa cells were harvested and washed twice with assay buffer (0.2% v/v FBS in live cell imaging solution). The cells were counted using 0.4% Trypan Blue solution and an automated cell counter, after which 1-3*10^5 cells were incubated with different concentrations of sfGFP(Y151pAzF)-SDF-1β, sfGFP or commercially available SDF-1β for 30 minutes in the dark at a final concentration of 12.5*10^5 cells/mL. For a competition experiment, 1 μM commercially obtained SDF-1β or 1 μM AMD3100 was co-incubated with 100 nM sfGFP(Y151pAzF)-SDF-1 β. Cells incubated with commercially available SDF-1β were washed with cold flow cytometry buffer, centrifuged at 1800 rpm for 3 minutes and stained with AlexaFluor647-conjugated-anti-human/mouse SDF-1 antibody (1000 nM; final volume 100 μL) for 30 minutes on ice protected from light. Following incubation, the cells were washed with cold assay buffer and centrifuged at 1800 rpm for 3 minutes. Cells were washed twice with assay buffer and fixated in a solution of 4% formaldehyde. Samples were analyzed by flow cytometry using a BD FACSAria III (BectonDickinson) and FACSDiva Software (Becton-Dickinson). sfGFP was excited at 488 nm and emission was recorded using a 530/30 emission filter. The AlexaFluor 647-conjugated antibody was excited at 633 nm and emission was recorded using a 660/20

emission filter. Single cells were gated based on their forward and sideward scatter. For each sample, >10,000 cells were recorded. The binding is always represented as a fold change in fluorescence compared to the untreated control, unless otherwise stated, with error bars representing the standard deviation.

Confocal microscopy

Images of HeLa cells were made using a Leica TCS SP5 confocal microscope equipped with an HCX PL Apo CS 63x/1.20 UV-VIS-IR water-immersion objective and HyD detector. The pinhole was set to 1 Airy Unit. Single plane images of 1024x1024 pixels were acquired with a scan rate of 200 Hz, and line averaged 6 times. sfGFP was excited at 490 nm and emission was measured between 500 and 600 nm.

Supporting figures

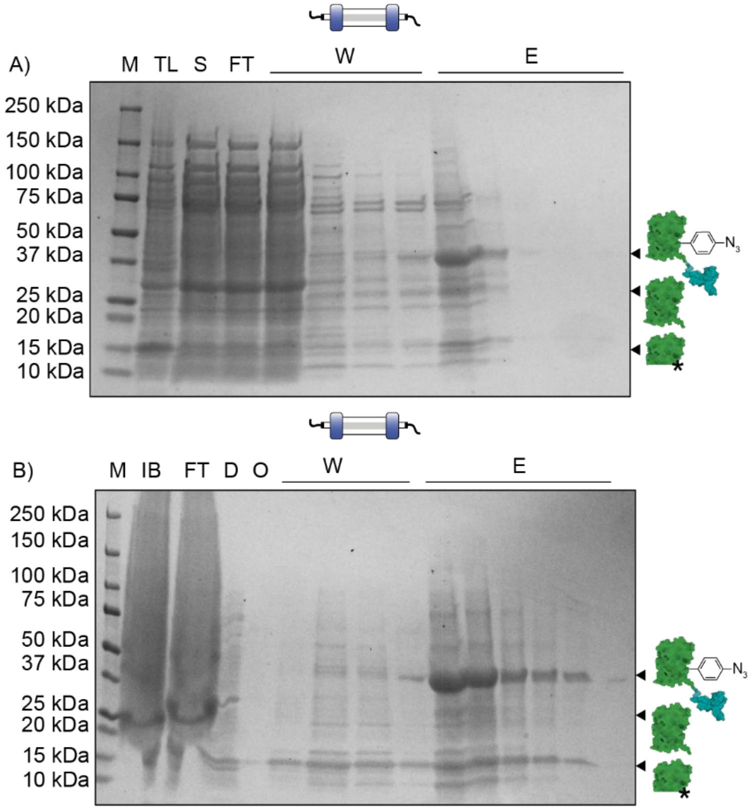

Figure S1. SDS-PAGE analysis of the purification of sfGFP(Y151pAzF)-SDF-1β following expression from BL21(DE3). (A) Uncropped SDS-PAGE gel of the Ni^{2+}-NTA purification of sfGFP(Y151pAzF)-SDF-1β expressed as soluble (S) protein. (B) Uncropped SDS-PAGE gel of the Ni^{2+}-NTA purification and on-column refolding of sfGFP(Y151pAzF)-SDF-1β expressed in inclusion bodies (IB). For the inclusion bodies, sfGFP(Y151pAzF)-SDF-1β was unfolded in 6M guanidinium hydrochloride and loaded onto a column containing Ni^{2+}-NTA-agarose resin. On the column, the majority of guanidinium hydrochloride was washed away in the flow through (FT) using a detergent (D) buffer containing Triton X-100 and 2-mercaptoethanol, allowing the protein to fold into its tertiary structure. Next, the disulfide bonds were formed by washing away the reducing agents using an oxidation (O) buffer containing reduced- and oxidized glutathione. The protein was then washed (W) with 30 mM imidazole and subsequently eluted (E) using 1 M imidazole. Different products are indicated with cartoons; sfGFP-SDF-1β (39.7 kDa), sfGFP (27.8 kDa) and truncation product sfGFP* (17.4 kDa). TL= total lysate.

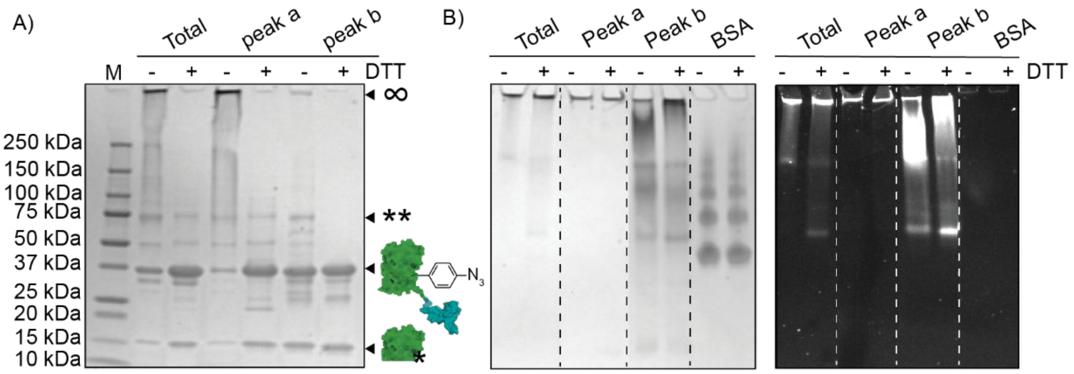

Figure S2. (A) SDS-PAGE and (B) native PAGE analysis of sfGFP(Y151pAzF)-SDF-1β SEC peaks under reducing (+DTT) and non-reducing (-DTT) conditions. The fluorescence signal of sfGFP (right) on native PAGE was measured before coomassie staining (left). Different species are indicated with cartoons; sfGFP-SDF-1β (39.7 kDa), truncation product sfGFP* (17.4 kDa). ∞ represents soluble aggregates. ** represents dimers.

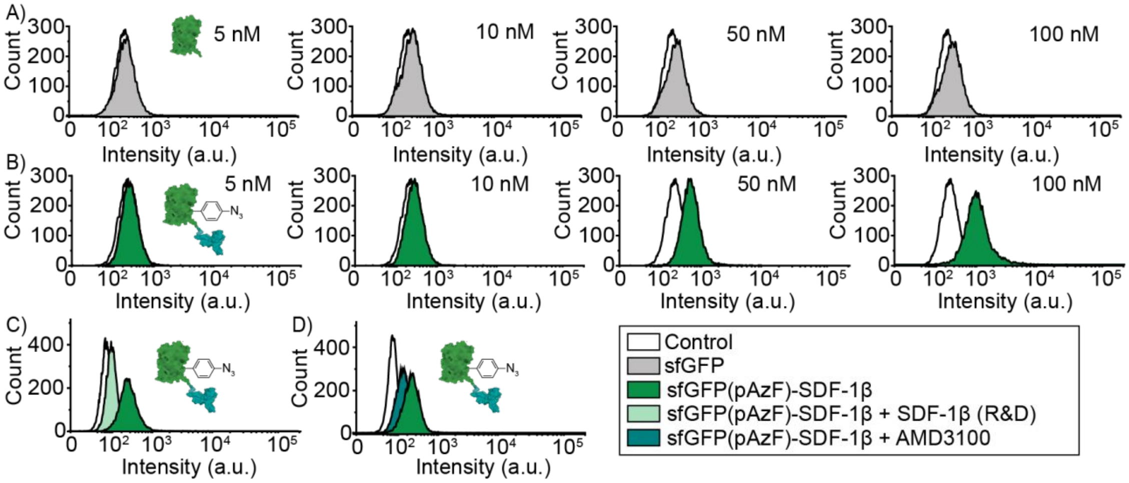

Figure S3. Representative histograms of the fluorescence of HeLa cells measured using flow cytometry after incubation with (A) different concentrations of sfGFP, (B) different concentrations of sfGFP sfGFP(Y151pAzF)-SDF-1β, (C) 100 nM sfGFP(Y151pAzF)-SDF-1β with and without 1μM commercial SDF-1β (R&D) (light green and dark green respectively) and (D) 100 nM sfGFP(Y151pAzF)-SDF-1β with and without 1μM AMD3100 (blue and dark green, respectively). White represents an untreated control. 10,000 cells were measured per sample.

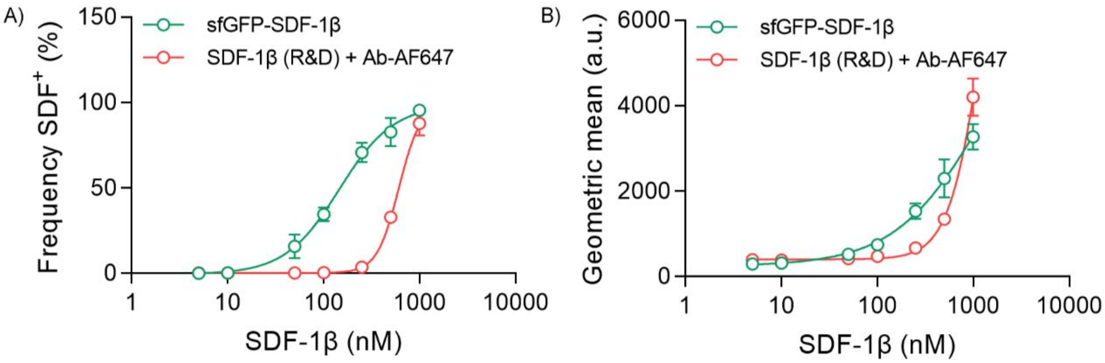

Figure S4. Flow cytometry analysis of the fluorescence of HeLa cells following incubation with different concentrations of sfGFP(Y151(pAzF)-SDF-1β (green) or commercial SDF-1β + AlexaFluor647-conjugated-anti-human/mouse SDF-1 antibody (Ab-AF647) (red) to the cell membrane of HeLa cells (A) Frequency of SDF-1β positive cells. (B) Mean fluorescence intensity of HeLa cells. n≥3. 10,000 cells were measured per sample. A sigmoidal four parameter logistic fit (4PL) was used

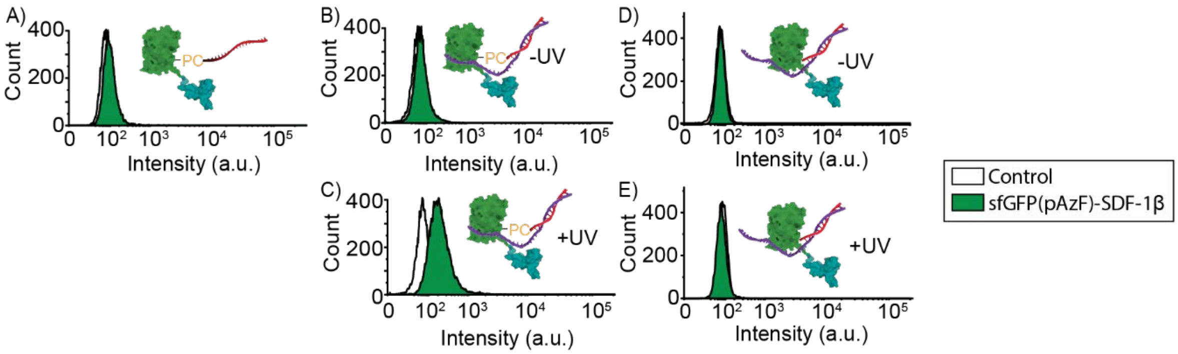

Figure S5. Representative histograms of the fluorescence of HeLa cells measured using flow cytometry. Cells were either incubated with 100 nM (A) 21nt-ssDNA-PC-sfGFP-SDF-1β in the dark, (B) 42nt+21nt-ssDNA-PC-sfGFP-SDF-1β in the dark, (C) 42nt+21nt-ssDNA-PC-sfGFP-SDF-1β previously exposed to UV, (D) 42nt+21nt-ssDNA -sfGFP-SDF-1β in the dark, and (E) 42nt+21nt-ssDNA -sfGFP-SDF-1β previously exposed to UV. 10,000 cells were measured per sample. PC stands for photocleavable linker.

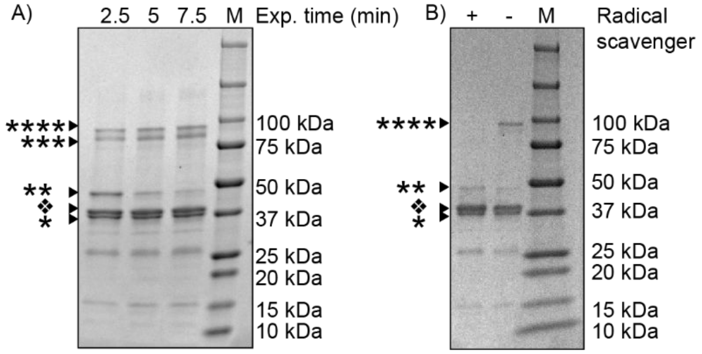

Figure S6. SDS-PAGE analysis of the photocleavage of the 21nt-ssDNA from sfGFP(Y151pAzF)-SDF-1β. (A) Cleavage of ssDNA from sfGFP(Y151pAzF)-SDF-1β with photocleavable (PC) linker following 2.5 up to 7.5 minutes of UV irradiation. (B) Cleavage in the absence and presence of 100 equivalents of the radical scavenger ascorbic acid. * Indicates the monomer sfGFP(Y151pAzF)-SDF-1β band, ❖ indicates a band just above the monomer, ** indicates the uncleaved 21nt-ssDNA-sfGFP-SDF-1β, ***/**** indicate possible dimer and trimer formation, respectively.

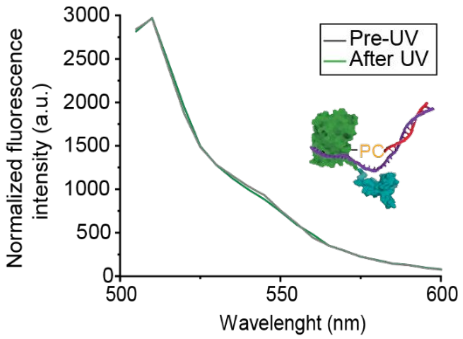

Figure S7. Fluorescence spectrum of 63nt-DNA-sfGFP-SDF-1β before and after 10 min UV irradiation. sfGFP was excited at 480 nm and emission was measured between 510 and 600 nm.

References

(1) Altenburg, W. J.; Yewdall, N. A.; Vervoort, D. F. M.; van Stevendaal, M. H. M. E.; Mason, A. F.; van Hest, J. C. M. Programmed Spatial Organization of Biomacromolecules into Discrete, Coacervate-Based Protocells. *Nat. Commun.* **2020**, *11* (1), 6282. https://doi.org/10.1038/s41467-020-20124-0.

(2) Young, T. S.; Ahmad, I.; Yin, J. A.; Schultz, P. G. An Enhanced System for Unnatural Amino Acid Mutagenesis in E. Coli. *J. Mol. Biol.* **2010**, *395* (2), 361–374. https://doi.org/10.1016/j.jmb.2009.10.030.

(3) Wang, L.; Schultz, P. G. A General Approach for the Generation of Orthogonal TRNAs. *Chem. Biol.* **2001**, *8* (9), 883–890. https://doi.org/10.1016/S1074-5521(01)00063-1.

(4) Veldkamp, C. T.; Peterson, F. C.; Hayes, P. L.; Mattmiller, J. E.; Haugner 3rd, J. C.; de la Cruz, N.; Volkman, B. F. On-Column Refolding of Recombinant Chemokines for NMR Studies and Biological Assays. *Protein Expr. Purif.* **2007**, *52* (1), 202–209. https://doi.org/10.1016/j.pep.2006.09.009.

(5) Lambert, T. J. FPbase: A Community-Editable Fluorescent Protein Database. *Nat. Methods* **2019**, *16* (4), 277–278. https://doi.org/10.1038/s41592-019-0352-8.
